# Axonal damage and inflammation response are biological correlates of decline in small-world values: a cohort study in autosomal dominant Alzheimer’s disease

**DOI:** 10.1093/braincomms/fcae357

**Published:** 2024-10-09

**Authors:** Lisa Vermunt, Courtney L Sutphen, Ellen Dicks, Diederick M de Leeuw, Ricardo F Allegri, Sarah B Berman, David M Cash, Jasmeer P Chhatwal, Carlos Cruchaga, Gregory S Day, Michael Ewers, Martin R Farlow, Nick C Fox, Bernardino Ghetti, Neill R Graff-Radford, Jason Hassenstab, Mathias Jucker, Celeste M Karch, Jens Kuhle, Christoph Laske, Johannes Levin, Colin L Masters, Eric McDade, Hiroshi Mori, John C Morris, Richard J Perrin, Oliver Preische, Peter R Schofield, Marc Suárez-Calvet, Chengjie Xiong, Philip Scheltens, Charlotte E Teunissen, Pieter Jelle Visser, Randall J Bateman, Tammie L S Benzinger, Anne M Fagan, Brian A Gordon, Betty M Tijms

**Affiliations:** Alzheimer center Amsterdam, Department of Neurology, Amsterdam Neuroscience, Programme Neurodegeneration, Amsterdam University Medical Centers, Vrije Universiteit, 1081 HZ Amsterdam, The Netherlands; Neurochemistry Laboratory, Departmentt of Laboratory Medicine, Amsterdam Neuroscience, Programme Neurodegeneration, Amsterdam University Medical Centers, Vrije Universiteit, 1081 HZ Amsterdam, The Netherlands; Washington University School of Medicine, St. Louis, MO 63110, USA; Alzheimer center Amsterdam, Department of Neurology, Amsterdam Neuroscience, Programme Neurodegeneration, Amsterdam University Medical Centers, Vrije Universiteit, 1081 HZ Amsterdam, The Netherlands; Department of Neurology, Mayo Clinic, Rochester, MN 55905, USA; Alzheimer center Amsterdam, Department of Neurology, Amsterdam Neuroscience, Programme Neurodegeneration, Amsterdam University Medical Centers, Vrije Universiteit, 1081 HZ Amsterdam, The Netherlands; Instituto de Investigaciones Neurológicas FLENI, Buenos Aires, Argentina; Department of Neurology, Alzheimer’s Disease Research Center, and Pittsburgh Institute for Neurodegenerative Diseases, University of Pittsburgh, Pittsburgh, PA 15213, USA; Dementia Research Centre, UCL Queen Square Institute of Neurology, London WC1N 3AR, UK; Department of Neurology, Massachusetts General Hospital, Boston, MA 02114, USA; Washington University School of Medicine, St. Louis, MO 63110, USA; Mayo Clinic Florida, Jacksonville, FL 32224, USA; Institute for Stroke and Dementia Research, University Hospital, Ludwig-Maximilian-University Munich, 81377 Munich, Germany; German Center for Neurodegenerative Diseases (DZNE), 37075 Göttingen, Germany; Department of Pathology and Laboratory Medicine, Indiana University, Indianapolis, IN 46202, USA; Dementia Research Institute at UCL, University College London Institute of Neurology, London W1T 7NF, UK; Department of Neurodegenerative Disease, Dementia Research Centre, London WC1N 3AR, UK; Section for Dementia Research, Hertie Institute for Clinical Brain Research and Department of Psychiatry and Psychotherapy, University of Tübingen, 72076 Tübingen, Germany; Mayo Clinic Florida, Jacksonville, FL 32224, USA; Washington University School of Medicine, St. Louis, MO 63110, USA; German Center for Neurodegenerative Diseases (DZNE), 37075 Göttingen, Germany; Section for Dementia Research, Hertie Institute for Clinical Brain Research and Department of Psychiatry and Psychotherapy, University of Tübingen, 72076 Tübingen, Germany; Washington University School of Medicine, St. Louis, MO 63110, USA; Neurologic Clinic and Policlinic, University Hospital and University Basel, 4031 Basel, Switzerland; German Center for Neurodegenerative Diseases (DZNE), 37075 Göttingen, Germany; Section for Dementia Research, Hertie Institute for Clinical Brain Research and Department of Psychiatry and Psychotherapy, University of Tübingen, 72076 Tübingen, Germany; German Center for Neurodegenerative Diseases (DZNE), 37075 Göttingen, Germany; Ludwig-Maximilians-Universität München, D-80539 München, Germany; Florey Institute, Melbourne, Parkville Vic 3052, Australia; The University of Melbourne, Melbourne, Parkville Vic 3052, Australia; Washington University School of Medicine, St. Louis, MO 63110, USA; Department of Clinical Neuroscience, Osaka City University Medical School, 558-8585 Osaka, Japan; Washington University School of Medicine, St. Louis, MO 63110, USA; Washington University School of Medicine, St. Louis, MO 63110, USA; German Center for Neurodegenerative Diseases (DZNE), 37075 Göttingen, Germany; Section for Dementia Research, Hertie Institute for Clinical Brain Research and Department of Psychiatry and Psychotherapy, University of Tübingen, 72076 Tübingen, Germany; Neuroscience Research Australia & School of Medical Sciences, NSW 2052 Sydney, Sydney, Australia; Barcelonaβeta Brain Research Center (BBRC), Pasqual Maragall Foundation, 08005 Barcelona, Spain; IMIM (Hospital del Mar Medical Research Institute), 08003 Barcelona, Spain; Servei de Neurologia, Hospital del Mar, 08003 Barcelona, Spain; Washington University School of Medicine, St. Louis, MO 63110, USA; Alzheimer center Amsterdam, Department of Neurology, Amsterdam Neuroscience, Programme Neurodegeneration, Amsterdam University Medical Centers, Vrije Universiteit, 1081 HZ Amsterdam, The Netherlands; Life Science Partners, 1071 DV Amsterdam, The Netherlands; Neurochemistry Laboratory, Departmentt of Laboratory Medicine, Amsterdam Neuroscience, Programme Neurodegeneration, Amsterdam University Medical Centers, Vrije Universiteit, 1081 HZ Amsterdam, The Netherlands; Department of Psychiatry and Neuropsychology, School for Mental Health and Neuroscience, Alzheimer Center Limburg, Maastricht University, 6229 ER Maastricht, Netherlands; Washington University School of Medicine, St. Louis, MO 63110, USA; Washington University School of Medicine, St. Louis, MO 63110, USA; Washington University School of Medicine, St. Louis, MO 63110, USA; Washington University School of Medicine, St. Louis, MO 63110, USA; Alzheimer center Amsterdam, Department of Neurology, Amsterdam Neuroscience, Programme Neurodegeneration, Amsterdam University Medical Centers, Vrije Universiteit, 1081 HZ Amsterdam, The Netherlands

**Keywords:** structural covariance network, autosomal dominant Alzheimer disease, axonal damage, neuronal injury, inflammation

## Abstract

The grey matter of the brain develops and declines in coordinated patterns during the lifespan. Such covariation patterns of grey matter structure can be quantified as grey matter networks, which can be measured with magnetic resonance imaging. In Alzheimer’s disease, the global organization of grey matter networks becomes more random, which is captured by a decline in the small-world coefficient. Such decline in the small-world value has been robustly associated with cognitive decline across clinical stages of Alzheimer’s disease. The biological mechanisms causing this decline in small-world values remain unknown. Cerebrospinal fluid (CSF) protein biomarkers are available for studying diverse pathological mechanisms in humans and can provide insight into decline. We investigated the relationships between 10 CSF proteins and small-world coefficient in mutation carriers (*N* = 219) and non-carriers (*N* = 136) of the Dominantly Inherited Alzheimer Network Observational study. Abnormalities in Amyloid beta, Tau, synaptic (Synaptosome associated protein-25, Neurogranin) and neuronal calcium-sensor protein (Visinin-like protein-1) preceded loss of small-world coefficient by several years, while increased levels in CSF markers for inflammation (Chitinase-3-like protein 1) and axonal injury (Neurofilament light) co-occurred with decreasing small-world values. This suggests that axonal loss and inflammation play a role in structural grey matter network changes.

## Teaser

Brain development and maturation leads to grey matter networks that can be measured using MRI. The biological mechanisms causing a loss of network integrity in Alzheimer are unknown. The results of Vermunt *et al*. suggest that axonal loss and inflammation play a role in structural grey matter network changes.

## Graphical Abstract

**Graphical abstract:**
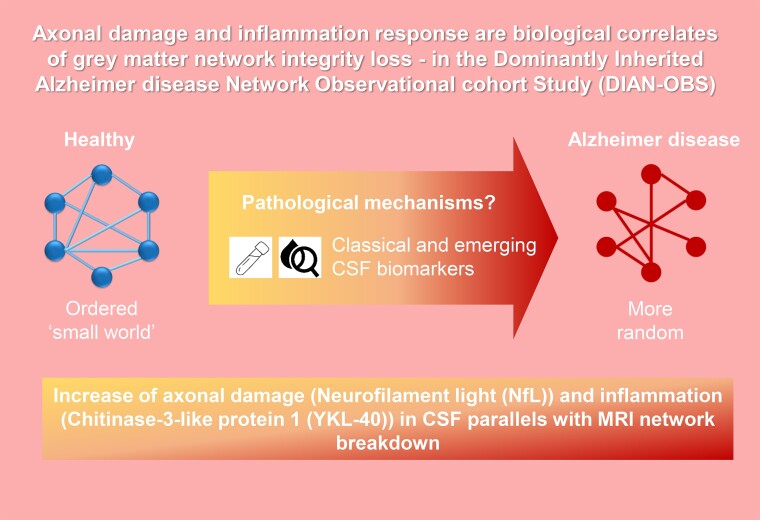

## Introduction

Brain areas implicated in similar functions show covariation in cortical morphology on magnetic resonance imaging (MRI), and these covariation patterns can be precisely quantified with a network approach.^1-3^ In neurodegenerative diseases, such as Alzheimer’s disease, grey matter networks become disrupted.^3-9^ With increasing disease severity in Alzheimer’s disease, grey matter networks become more randomly organized, as indicated by a lower small-world coefficient.^9-11^ More specific, the small-world coefficient is a summary measure that reflects the balance of local information processing, as indicated by the clustering coefficient, over information integration between clusters, as indicated by the path length.^12^ In Alzheimer’s disease, the small-world coefficient decreases in value with worse cognitive impairment, and in predementia stages, lower values are predictive for steeper cognitive decline over time.^10,13-17^ Similar alterations have been observed in sporadic and autosomal dominant Alzheimer’s disease (ADAD).^11^ Loss of small-world values can already be detected in cognitively normal individuals with amyloid-β (Aβ) aggregation.^10,18,19^ Still, the biological mechanisms that explain the deterioration of network organization remain unclear. Changes in grey matter networks could result from multiple pathophysiological processes such as synaptic dysfunction and loss, axonal degeneration, neuronal loss, and local swelling in response to infiltration of inflammatory cells. A better understanding of network disruptions over the course of Alzheimer’s disease may inform new hypotheses regarding what biological process maintain brain connectivity and preserve cognitive function.

In cerebrospinal fluid (CSF), proteins can be measured that reflect ongoing biological processes in the brain. CSF biomarkers are used for the biological definition of Alzheimer’s disease based on abnormal concentrations of Aβ (Aβ42/40 ratio), hyperphosphorylation of tau (181-phosphorylated fraction [pTau]) and neuronal injury (total tau [tTau]).^20^ In addition to these core Alzheimer’s disease measures, other biomarkers have robustly been related to Alzheimer’s disease, and provide information on additional pathological brain alterations occurring in the disease:^21^ Increased levels of synaptosomal-associated protein-25 (SNAP-25) and neurogranin (Ng) levels are markers of pre-synaptic and post-synaptic dysfunction, respectively; visinin-like protein 1 (VILIP-1) a calcium-sensor protein increased with neuronal injury; and neurofilament light chain (NfL) of neuroaxonal damage.^22-27^ In addition, chitinase-3-like protein 1 (YKL-40), an inflammation marker, and soluble TREM2 (sTREM2)^23,24,27-30^ a marker of microglia, are also elevated in Alzheimer’s disease and provide insight into inflammatory processes. These markers have been associated with Alzheimer’s disease, but it remains unknown if they may influence grey matter networks.

We investigated this question in carriers of ADAD genetic mutations. This allows for investigation of the disease processes before onset of symptoms due to relatively conserved dementia onset age and few age-related co-pathologies.^31^ We assessed the associations between both the core and emerging CSF biomarkers for Alzheimer’s disease and the individual grey matter network summary statistic, the ‘small-world coefficient’ as this metric has been robustly associated with cognitive decline in Alzheimer’s disease across multiple studies.^8,11,16,17,32^ We also tested *within* the mutation carriers whether the relationships observed were dependent on disease stage. For this step, the mutation carriers were divided into four disease severity groups: (1) normal CSF ratio of pTau/Aβ_42_ (indicating absence of underlying brain amyloid); (2) abnormal CSF ratio and clinical dementia rating (CDR) 0 (no impairment); (3) abnormal CSF ratio and CDR 0.5 (very mild Alzheimer’s disease dementia); (4) abnormal CSF ratio and CDR 1–3 (mild to severe Alzheimer’s disease dementia).

## Materials and methods

### Participants and design

Data were obtained from the Dominantly Inherited Alzheimer Network Observational Study (DIAN-Obs).^33^ For the DIAN study, ADAD mutation carriers (MC) in the presenilin 1 [*PSEN1*], presenilin 2 [*PSEN2*] and amyloid precursor protein genes and their non-carrier (NC) family members undergo longitudinal clinical and cognitive examinations, neuroimaging and biospecimen donations. We evaluated data that passed quality control and was included in data freeze 12. Families with Flemish and Dutch mutations were excluded from analyses because these mutations result in a different phenotype, with primarily cerebral amyloid angiopathy. The study was approved by the ethical review board at Washington University, St. Louis, Missouri, USA and local IRBs. The estimated years to symptom onset (EYO) for each individual was defined as the mutation-specific mean age at onset subtracted from the individuals’ visit age (e.g. for the *PSEN1* G206A mutation, the mean age at onset is 53).^31^ Where the mutation age of onset was unknown, the family-specific parental age of disease onset was used instead. For example, if the mean age at symptom onset is 53 years for a specific family mutation, then a 43-year-old individual, regardless of mutation status, would have an EYO of −10. This indicates an individual with the mutation is expected to show clinical symptoms of Alzheimer’s disease 10 years later and allows comparison of biomarkers with the NCs on the same timeline, as well as between MCs and NCs from different families and mutations. For the biomarker comparisons, we selected the first visit at which individuals had both CSF and MRI data available.

### Group definitions

Participants were stratified in two ways. The first set of analyses focused on comparing all MCs to their familial NC controls. In the second set of analyses, MCs were split into four disease stage groups based upon their biomarker status and clinical dementia rating (CDR).^34^ Group 1 had a normal CSF ratio of pTau/Aβ_42_ (<0.019^35^) (indicating absence of underlying brain amyloid). Groups 2–4 had abnormal ratios (indicating presence of amyloid) and increasing CDRs of: Group 2: CDR = 0, no impairment; Group 3: CDR = 0.5, very mild dementia; Group 4: CDR ≥ 1 mild to severe dementia.

### MR preprocessing

MR scans were collected and preprocessed according to the protocols of the Alzheimer’s Disease Neuroimaging Initiative (1.1 by 1.1 by 1.2 mm^3^ voxels, repetition time = 2300, echo time = 2.95, flip angle 9°), described in detail in.^36-38^ For the network extractions, T1-weighted scans were first segmented into grey matter, white matter and CSF in native space with Statistical Parametric Mapping 12 (SPM12; Wellcome Trust Centre for Neuroimaging, UCL Institute of Neurology, London, UK). The segmentations were checked and resliced into 2 mm by 2 mm by 2 mm voxels, and this was the input for the grey matter network extraction.

### Calculation of grey matter network metrics

Single-subject grey matter network metrics were extracted from preprocessed grey matter segmentations according to previously published procedures (https://github.com/bettytijms/Single_Subject_Grey_Matter_Networks, see also ref. 2), in brief: Grey matter segmentations were parcellated into cubes of 3 by 3 by 3 voxels, which formed the nodes of the network. The Pearson’s correlation coefficient was then calculated for grey matter intensities across the voxels for each pair of cubes. Next, the correlation matrices were binarized by retaining only correlations that remained significant, using a person specific threshold that corrected *P*-values for multiple comparisons by permutation.^2^ Briefly, for each individual we removed spatial information from the MRI scan, while leaving intact the statistical distributions of grey matter intensity values, and recomputed the correlations between network nodes, providing a null distribution in which the correlations reflect chance level associations. The level for significance was determined by determining the level of correlations of which only 5% of values from the distribution exceeded.^39^ This approach ensures that individuals do not differ in the amount of spurious correlations included in the network, which can influence subsequent network metric values.^40^ Finally, we calculated the small-world metric for each network with the brain connectivity toolbox (https://sites.google.com/site/bctnet/^41^) modified for large sized networks. The small-world coefficient is a whole-brain network summary statistic that reflects the balance of localized information processing within specialized modules, as quantified by the clustering coefficient, together with information exchange between clusters along long range connections, as quantified by a short path length. In networks that have a ‘small-world’ property, the network has a more clustered structure than a random network, while the path length is similar to that of a random network.^12,42^ This is quantified by normalizing the normalized clustering coefficient by the clustering coefficient of a random network, and to divide this metric by path length that is normalized by the path length of a random network. In this study, we repeated randomization of person specific networks 5 times. Small-world values larger than 1 indicate more structure than a random network organization.^42,43^

### Cerebrospinal fluid markers

Participants underwent lumbar puncture after overnight fasting. Samples were collected via gravity drip in polypropylene tubes and sent on dry ice to the DIAN biomarker laboratory at Washington University. The samples were then thawed and aliquoted (0.5 mL) in polypropylene tubes, stored at −84°C before measurements of SNAP-25, Ng, VILIP-1 and YKL-40. Additional aliquots of each sample were shipped on dry ice for the measurements of Aβ_40_, Aβ_42_, pTau and tTau by the Shaw laboratory at the University of Pennsylvania,^44^ of NfL by the Kuhle laboratory in Basel,^45^ and of sTREM2 by the Haass laboratory in Mϋnich.^28,30^ For details on the protocols, see.^25,26,28,46^ Briefly, Aβ_40_, Aβ_42_, pTau and tTau levels were determined using the automated Elecsys assay, and values of Aβ_40_ and Aβ_42_ outside the measurement ranges were extrapolated on the calibration curve.^46^ SNAP-25, Ng and VILIP-1 were measured with antibodies developed in the laboratory of Dr. Jack Ladenson at Washington University in St. Louis, as part of micro-particle-based immunoassays using the Singulex (now part of EMD Millipore; Alameda, CA, USA) Erenna system.^25^ YKL-40 was measured with plate-based enzyme-linked immunoassay (MicroVue ELISA; Quidel, San Diego, CA, USA).^25^ NfL was measured on a single-molecule array using the capture monoclonal antibody 47:3 and biotinylated detection antibody 2:1 (UmanDiagnostics AB, Sweden).^26^ Soluble TREM2 (sTREM2) was measured using the Meso Scale Discovery platform with an in-house developed ELISA based on commercially available antibodies.^28,47^ The sTREM2 concentrations are reported relative to an internal standard sample that was loaded onto each assay plate as a way to account for inter-plate variation. Missingness of biomarker levels in part of the individuals, due to not all samples being measured as part of each of the biomarker projects, was regarded as missing at random, meaning that the statistical analyses were conducted with the available data.

### Statistical analysis

In all linear models tTau, pTau, SNAP-25, Ng, VILIP-1, YKL-40, NfL and sTREM2 were log-transformed to approach normality. There was one outlier for SNAP-25, but it did not influence the effect sizes nor significance, so we did not exclude any samples from the analyses. To aid comparability of slope estimates, the variables were Z-transformed according to the entire cohort. We tested the associations between the CSF biomarkers as predictors and the small-world coefficient as the outcome with three linear regression models. Model 1 was adjusted for sex (R code: small-world index ∼ log_CSF_biomarker + sex, data = data); Model 2 also included a fixed term for mutation status and its interaction with the predictor (R code: small-world index ∼ log_CSF_biomarker * mutation + sex, data = data); Model 3 had additional adjustment for age effects as main effect (R code: small-world index ∼ log_CSF_biomarker * mutation + sex + age, data = data). The age adjustment model was not the primary model, because the association between age and disease progression within the mutation carriers means that correction for age in a DIAD group lead to a correction for disease. At the same time, we were inclined to deeper investigate if possible age associations and which part of the effects, in the NC in particular, might be explained by ageing processes. We also tested the Spearman correlations between the biomarkers (Supplementary Fig. 1). We further performed a subgroup analysis *within* MCs only so to investigate disease stage effects: these models included the CSF biomarkers as predictor, a fixed term for the interaction between the severity groups and their predictor. In cases of significant interactions terms, we ran *post hoc* pairwise comparisons using the Tukey HSD procedure. The same approach was applied to detect possible differences between the mutation types in DIAN (PSEN1, PSEN2 and APP). Lastly, we estimated trajectories for all markers studied by EYO, using a previously developed Bayesian inference linear mixed effect model^37,48^ to obtain insight into the relative ordering of biomarker trajectories. The statistical model to fit biomarker trajectories by EYO accounted for non-linear effects by using a restricted cubic spline to model EYO, with knots on the 0.1, 0.5 and 0.9 of the distribution. The models had fixed terms for EYO, mutation status, their interaction and a random effect for family cluster. Models were adjusted for sex and for the small-world coefficient additionally for total grey matter volume. For the trajectories, we used the biomarker data of the first available visit (Supplementary Table 1). Model parameters were estimated with Hamiltonian Markov chain Monte Carlo sampling of the posterior distribution, with cauchy prior, 10 000 iterations in 8 chains, and thinning of 10 in the STAN and rstanarm package for R (R code: model_fit <- stan_glmer(standardized_biomarker_value ∼ (1 | family_id) + eyo_1 + eyo_2 + mutation_status + eyo_term_1*mutation_status + eyo_term_2*mutation_status + covariates, data = data, family = gaussian(), prior = cauchy(), prior_intercept = cauchy(), chains = 8, cores = 1, iter = 10000, thin = 10)**).** The EYO point of divergence is when the 99% credible intervals of the difference distribution between MCs and NCs did not overlap 0. We also provide the 95% and 99.5% of the credible intervals. Before fitting this model, the CSF and MRI biomarkers were Z-scored to young NCs (<40 years old, *n* = 81, Table 1). All statistical analyses were conducted in R (version 3.5.3) using the emmeans. car, lmer, rstan and rstanarm-packages.^49^

**Table 1:** Demographics and baseline summary data on predictors and outcomes

	Non-carriers (NCs)		Mutation carriers (MCs)				
	All (*n* = 136)	NCs <40 years old (*n* = 81)	All (*n* = 216)	MCs: ratio neg (*n* = 84)	MCs: CDR 0, ratio pos (*n* = 63)	MCs: CDR 0.5, ratio pos (*n* = 43)	MCs: CDR 1–3, ratio pos (*n* = 26)
Demographics							
*N* (%) Male	53 (39%)	32 (40%)	96 (44%)	35 (42%)	28 (44%)	19 (44%)	14 (54%)
Age, years	38 ± 12	31 ± 6	39 ± 10	32 ± 8	38 ± 9	47 ± 9	47 ± 9
EYO, years	−10 ± 12	−17 ± 9	−9 ± 11	−17 ± 8	−8 ± 7	1 ± 6	4 ± 4
PSEN1/PSEN2/APP, %	NA	NA	77/8/15	67/11/22	84/10/6	79/5/16	88/0/12
Years of education, median ± IQR	15 ± 3	15 ± 2	14 ± 4	15 ± 3	15 ± 4	14 ± 4	12 ± 2
CDR (0/0.5–1/2–3), *N*	131/5/0	0/2/0	142/66/8	79/5/0	63/0/0	0/43/0	0/18/8
MMSE, median ± IQR	30 ± 1	30 ± 1	29 ± 3	29 ± 1	29 ± 2	26 ± 4	16 ± 10
Grey matter network							
Small-world coefficient	1.62 ± 0.05	1.65 ± 0.05	1.59 ± 0.08	1.64 ± 0.06	1.60 ± 0.05	1.55 ± 0.08	1.46 ± 0.07
Traditional CSF markers							
Aβ_42_ pg/ml	1407 ± 466	1292 ± 442	974 ± 634	1526 ± 655	716 ± 279	553 ± 208	510 ± 217
Aβ_40_ pg/ml	15 698 ± 4418	14 398 ± 4204	14 862 ± 4760	15 607 ± 5080	15 004 ± 4749	14 483 ± 4114	12 741 ± 4241
pTau pg/ml	14 ± 5	13 ± 4	31 ± 23	14 ± 4	32 ± 18	46 ± 23	57 ± 28
tTau pg/ml	169 ± 55	154 ± 49	290 ± 162	177 ± 48	305 ± 120	375 ± 142	475 ± 241
Ratio aβ_42/40_	0.089 ± 0.01	0.089 ± 0.007	0.066 ± 0.035	0.099 ± 0.031	0.049 ± 0.017	0.039 ± 0.012	0.042 ± 0.015
ratio Aβ_42/40_ ↓0.075, *N* (%)	6 (4%)	1 (1%)	144 (67%)	19 (23%)	58 (92%)	42 (98%)	25 (96%)
ratio pTau/Aβ_42_	0.010 ± 0.004	0.010 ± 0.002	0.052 ± 0.053	0.010 ± 0.004	0.051 ± 0.034	0.091 ± 0.049	0.120 ± 0.065
ratio pTau/Aβ_42_ ↑0.0198, *N* (%)	2 (1%)	0 (0%)	132 (61%)	-	-	-	-
Emerging CSF markers							
SNAP-25 pg/ml	3.6 ± 1.3	3.2 ± 1.1	4.6 ± 1.9	3.6 ± 1.1	4.5 ± 1.5	5.2 ± 1.7	6.4 ± 2.7
Ng pg/ml	1563 ± 741	1447 ± 765	2297 ± 1212	1638 ± 682	2526 ± 1109	2673 ± 1164	3120 ± 1748
NfL pg/ml	793 ± 544	564 ± 396	1939 ± 1762	531 ± 190	1033 ± 650	2630 ± 1643	3873 ± 1657
VILIP-1 pg/ml	133 ± 50	122 ± 48	174 ± 79	135 ± 47	179 ± 71	198 ± 75	236 ± 114
YKL-40 ng/ml	133 ± 66	98 ± 37	173 ± 88	109 ± 37	169 ± 69	229 ± 81	280 ± 89
sTREM2, relative to reference sample	0.47 ± 0.22	0.43 ± 0.21	0.58 ± 0.29	0.42 ± 0.15	0.48 ± 0.28	0.73 ± 0.3	0.74 ± 0.3

MC, mutation carrier; EYO, estimated years to symptom onset; CDR, clinical dementia rating scale; Aβ, Amyloid beta; pTau, phosphorylated Tau; tTau, total Tau; SNAP-25,Synaptosomal-Associated Protein 25 kDa; Ng, Neurogranin; NfL, Neurofilament Light; VILIP-1, visinin-like protein 1; YKL40, Chitinase 3-like 1; sTREM2, soluble TREM2 relative to a reference sample. CSF biomarkers not available for the whole sample: SNAP (*n* = 330), Ng (*n* = 331), VILIP1 (*n* = 330), YKL40 (*n* = 331), NfL (*n* = 165), sTREM2 (*n* = 164).

## Results

The presented analyses included 219 MCs and 136 NCs (age mean ± SD 39 ± 11; EYO mean ± SD −9 ± 11). In the MC group, 84 (39%) individuals had normal CSF ratio pTau/Aβ_42_. Among MCs with an abnormal CSF pTau/Aβ_42_ ratio, 63 (29%) individuals had CDR 0, 43 (20%) CDR 0.5 and 26 (12%) CDR 1–3. The group characteristics are shown in Table 1.

### Associations between CSF biomarkers and the small-world coefficient

**Figure 1:**
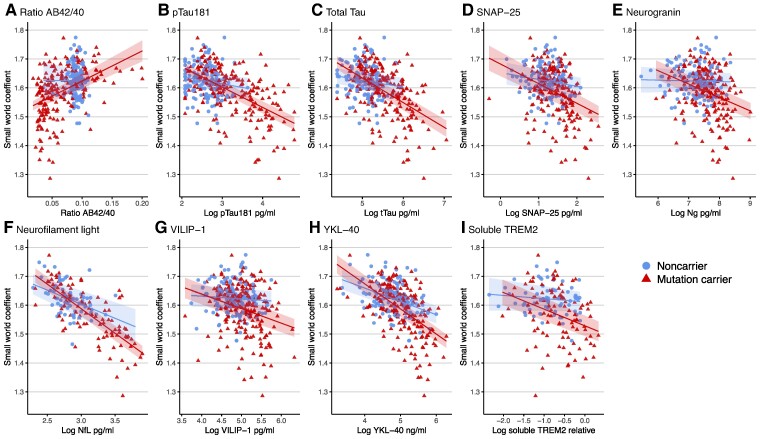
**Associations between CSF biomarkers and grey matter networks for mutation carriers and non-carriers.** Linear models predicting the small-world coefficient with the respective CSF biomarkers, adjusted for sex. All biomarkers were significantly associated to the small-world coefficient (*P* < 0.05). We tested the interaction with mutation status. All of the interactions, except for the AB42/40 ratio and sTREM2, were significant (*P* < 0.05), meaning that the strength of the relationship between the CSF biomarker and the grey matter network value dependent on mutation status. See for the test details in Table 2. The graphs show the predicted values with 95% confidence intervals. Each panel (**A–I**) represents a biomarker. Aβ, Amyloid beta; pTau, phosphorylated Tau; tTau, total Tau; SNAP-25, Synaptosomal-Associated Protein 25 kDa; Ng, Neurogranin; NfL, Neurofilament Light; VILIP-1, visinin-like protein 1; YKL40, Chitinase 3-like 1; sTREM2, soluble TREM2 relative to a reference sample.

**Figure 2:**
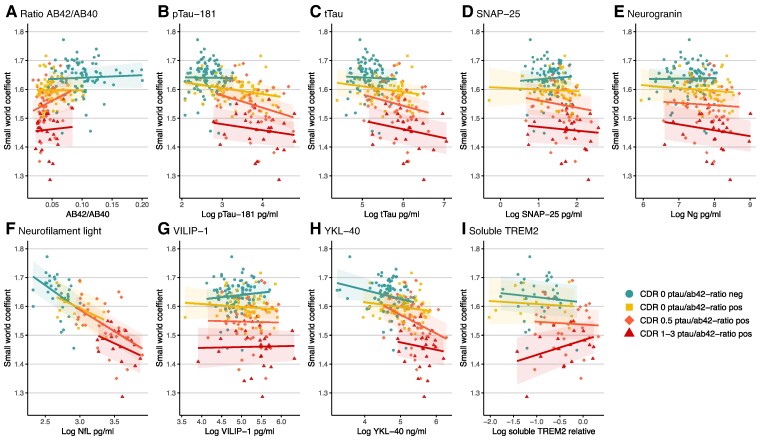
**Associations between CSF biomarkers and grey matter networks within mutation carriers by disease stage.** Linear model predicting the small-world coefficient with the respective CSF biomarkers, adjusted for sex. We tested the interaction with disease severity within the mutation carriers. None of the interactions with mutation type are significant (<0.05). The graphs show the predicted values with 95% confidence intervals. See Supplementary Table 2 for test statistics. Each panel (**A–I**) represents a biomarker. Aβ, Amyloid beta; pTau, phosphorylated Tau; tTau, total Tau; SNAP-25, Synaptosomal-Associated Protein 25 kDa; Ng, Neurogranin; NfL, Neurofilament Light; VILIP-1, visinin-like protein 1; YKL40, Chitinase 3-like 1; sTREM2, soluble TREM2 relative to a reference sample.

Across the whole group, we found that all Alzheimer’s disease markers were related to alterations in grey matter networks (Table 2). Higher levels of NfL were most strongly related to lower small-world values (β = −0.72 [95% CI −0.83, −0.61]; *P* < 0.001), followed by YKL-40 (β = −0.53 [95% CI −0.63, −0.44]; *P* < 0.001), and pTau (β = −0.53 [95% CI −0.61, −0.44]; *P* < 0.001, Table 2). Repeating analyses including an interaction term for mutation type with the CSF markers on small-world values indicated that relationships did not differ between mutation types (APP, PSEN1 or PSEN2, all *P*-values interactions >0.05; Supplementary Fig. 2). *Post hoc*, we tested for the strongest correlated marker, NfL, we then studied if including YKL-40 or pTau-181, in the model explained more variance in grey matter networks. We found that no extra variance was explained and only the effect of NfL on lower small-world integrity remained. Models taking into account interaction terms for mutation status and CSF predictor were significant for SNAP-25, Ng, pTau, tTau, NfL, VILIP-1 and YKL-40 (*P* < 0.05, Fig. 1). *Post hoc* comparisons showed that higher levels of SNAP-25 (−0.37 [95% CI −0.50, −0.24]) and Ng (−0.35 [95% CI −0.48, −0.21]), pTau (−0.58 [95% CI −0.69, −0.48]), tTau (−0.55 [95% CI −0.67, −0.44]) and VILIP-1 (−0.29 [95% CI −0.42, −0.16]) were related to lower small-world values specifically in MCs. The association of higher NfL and YKL-40 and lower small-world values was observed in both MCs and NCs, and this was stronger in MCs for YKL-40 (NfL: MC = −0.76 [95% CI −0.89, −0.64] and NC = −0.44 [95% CI −0.77, −0.17]; YKL-40: MC = −0.61 [95% CI −0.72, −0.49] and NC = −0.32 [95% CI −0.48, −0.17]). When repeating models correcting for age, interaction effects for mutation status changed by 0.14–0.39 and remained significant for SNAP-25, Ng, pTau, tTau, NfL and YKL-40 (*P* < 0.05), but evidence for VILIP-1 is weaker (*P* = 0.08). Next, we studied in MCs whether the observed associations were specific to disease stage (Supplementary Table 2, Fig. 2). No significant interaction terms with disease stage were observed, suggesting that associations of biomarkers and small-world values were not specific to a certain stage. Supplementary Figs 3–12 provide visual presentations the associations of all CSF markers with other network metrics (average degree, normalized clustering and normalized path length), as well as traditional structural MR measures.

**Table 2:** Associations between CSF markers and the small-world coefficient

	Model 1 Predictor	Model 2 Mutation status* Predictor			Model 3 Mutation status* Predictor and adjustment for age		
Predictors	Predictor (beta)	Interaction (t)	Non-carriers (est slope)	Carriers (est slope)	Interaction (t)	Non-carriers (est adj slope)	Carriers (est adj slope)
Aβ_42/40_ ratio	0.43 (0.33,0.52); ***P* < 0.001**	1.8; *P* = 0.075	−0.01 (−0.47, 0.45); *P* = 0.957	0.42 (0.31,0.52); ***P* < 0.001**	1.9; *P* = 0.064	−0.18 (−0.57, 0.22); *P* = 0.374	0.20 (0.11,0.30); ***P* < 0.001**
pTau-181	−0.53 (−0.61, −0.44); ***P* < 0.001**	−2.5; ***P* = 0.014**	−0.21 (−0.49, 0.07); *P* = 0.144	−0.58 (−0.69, −0.48); ***P* < 0.001**	−2; ***P* = 0.047**	0.06 (−0.18, 0.31); *P* = 0.605	−0.39 (−0.49, −0.29); ***P* < 0.001**
tTau	−0.48 (−0.57, −0.39); ***P* < 0.001**	−3.1; ***P* = 0.002**	−0.16 (−0.38, 0.07); *P* = 0.167	−0.55 (−0.67, −0.44); ***P* < 0.001**	−2.6; ***P* = 0.010**	0.05 (−0.14, 0.24); *P* = 0.596	−0.36 (−0.46, −0.26); ***P* < 0.001**
SNAP-25	−0.33 (−0.43, −0.22); ***P* < 0.001**	−2.1; ***P* = 0.035**	−0.13 (−0.31, 0.05); *P* = 0.162	−0.37 (−0.50, −0.24); ***P* < 0.001**	−2.2; ***P* = 0.026**	0.04 (−0.12, 0.19); *P* = 0.645	−0.14 (−0.25, −0.02); ***P* = 0.019**
Ng	−0.28 (−0.38, −0.17); ***P* < 0.001**	−2.9; ***P* = 0.004**	−0.02 (−0.20, 0.16); *P* = 0.836	−0.35 (−0.48, −0.21); ***P* < 0.001**	−2.7; ***P* = 0.008**	0.07 (−0.07, 0.22); *P* = 0.317	−0.20 (−0.31, −0.09); ***P* = 0.001**
NfL	−0.72 (−0.83, −0.61); ***P* < 0.001**	−2.2; ***P* = 0.032**	−0.44 (−0.71, −0.17); ***P* = 0.002**	−0.76 (−0.89, −0.64); ***P* < 0.001**	−3.7; ***P* < 0.001**	0.01 (−0.30, 0.32); *P* = 0.940	−0.53 (−0.68, −0.38); ***P* < 0.001**
VILIP-1	−0.26 (−0.37, −0.16); ***P* < 0.001**	−2.0; ***P* = 0.046**	−0.05 (−0.24, 0.14); *P* = 0.574	−0.29 (−0.42, −0.16); ***P* < 0.001**	−1.9; *P* = 0.060	0.07 (−0.09, 0.22); *P* = 0.401	−0.10 (−0.21, 0.01); *P* = 0.078
YKL-40	−0.53 (−0.63, −0.44); ***P* < 0.001**	−2.9; ***P* = 0.004**	−0.32 (−0.48, −0.17); ***P* < 0.001**	−0.61 (−0.72, −0.49); ***P* < 0.001**	−3.6; ***P* < 0.001**	0.05 (−0.11, 0.22); *P* = 0.534	−0.30 (−0.43, −0.18); ***P* < 0.001**
sTREM2	−0.33 (−0.50, −0.16); ***P* < 0.001**	−2; *P* = 0.052	−0.08 (−0.33, 0.17); *P* = 0.542	−0.40 (−0.61, −0.19); ***P* < 0.001**	−1.3; *P* = 0.195	0.13 (−0.08, 0.34); *P* = 0.229	−0.01 (−0.20, 0.18); *P* = 0.912

Aβ, Amyloid beta; pTau, phosphorylated Tau; tTau, total Tau; SNAP-25,Synaptosomal-Associated Protein 25 kDa; Ng, Neurogranin; NfL, Neurofilament Light; VILIP-1, visinin-like protein 1; YKL40, Chitinase 3-like 1; sTREM2, soluble TREM2 relative to a reference sample. Results of linear models predicting the small-world coefficient with the respective CSF biomarkers, adjusted for sex. In the second and third model, we tested the interaction with mutation status. All CSF markers, except the Aβ_42/40_ ratio are log-transformed. Significance at *P* < 0.05; Bonferroni corrected significance of nine tests is reached at *P* < 0.006. Significant values are presented in bold.

### Small-world coefficient and CSF biomarker trajectory by EYO

Finally, we estimated trajectories for all CSF and structural MRI markers according to EYO for the MCs, NCs and the difference between MCs and NCs (Supplementary Fig. 3; Supplementary Tables 3 and 4). Biomarker trajectories of the Aβ_42/40_ ratio, pTau, tTau, SNAP-25, Ng and VILIP-1 levels were different in MCs as compared to NCs before differences were observed in grey matter networks. NfL and YKL-40 trajectories were abnormal around the same time as small-world values, and sTREM2 and Aβ_40_ showed abnormal levels in MCs compared to NCs later than small-world values.

## Discussion

The main finding of our study is that CSF pathologic biomarkers showed associations with alterations in grey matter networks, and that axonal damage as measured with NfL showed the strongest relationship with worse grey matter network disruptions. Increased concentrations of the CSF markers for hyperphosphorylation of tau (pTau), neuronal injury and death (tTau and VILIP-1) and specific synaptic injury (SNAP-25 and Ng) were related to worse grey matter network organization in the MCs only. The observed associations were not dependent on staging based on a combination of the pTau/Aβ_42_ ratio and the global CDR, suggesting that they were similar across disease development. Most CSF biomarkers showed abnormal levels before grey matter network abnormality in the MCs compared to the NCs, and for NfL and YKL-40 the timing was closest aligned to grey matter network alterations.

To date, only the role of Aβ aggregation and tau had been studied in relation to decline in small-world values in individuals with Alzheimer’s disease.^18,19,32^ Those findings suggested that grey matter networks are sensitive to brain structural changes related to amyloid and tau aggregation in sporadic Alzheimer’s disease. Here, we found also that lower Aβ_42/40_ ratios were associated with grey matter network disruptions. We further detected relationships between markers of other pathological processes in ADAD and grey matter network disruptions. The most pronounced association was observed for NfL, which suggests that loss of axonal integrity plays a critical role in the loss of grey matter network organization. The link between axonal tract damage and deterioration of grey matter covariance in Alzheimer’s disease supports the idea that grey matter covariance networks reflect, at least in part, axonal connectivity.

We also observed that higher levels of the synaptic markers (SNAP-25 and Ng), pTau and neuronal damage (tTau and VILIP-1) were associated with grey matter network disruption, and this was specific for MCs. Synaptic maturation and co-activation are associated with grey matter covariance during development, promoting brain connectivity.^3^ In Alzheimer’s disease, synaptic damage in neurodegeneration could possibly influence brain connectivity in the opposite way. A study by Pereira *et al.*^50^ showed that synaptic markers like SNAP-25 and Ng were associated with worse functional connectivity in the default mode network and worse memory which supports this hypothesis. The biomarker trajectories suggest that synaptic damage and neuronal loss precedes the changes we observe with MRI, which could be a downstream effect. Recent analyses had already demonstrated that CSF pTau and tTau increase very early in the course of ADAD, in a more parallel fashion with amyloid aggregation than according to hypothetical models.^35,51^ Our findings suggest that loss of connectivity structures at the microscale (in neurons), could lead to disrupted whole-brain connectivity as measured on MRI. Longitudinal studies are needed to further examine the temporal relationship of these processes in more detail.

The associations between increased NfL and YKL-40, and network disruptions were also observed in NCs which suggests an effect of aging or another non-Alzheimer’s disease process. Age-specific reference intervals for NfL indicate that NfL starts to increase from 18 years of age with ±4% per year.^52,53^ For YKL-40 an ageing effect from adulthood is more speculative, because there are no reference intervals. Previous studies have shown that during aging NfL and YKL-40 levels increase and grey matter network measures decline, additive to elevations in predementia Alzheimer’s disease.^26,27,54-57^ Our finding suggests that also in non-Alzheimer’s disease related aging, loss of axonal integrity and inflammation may affect grey matter network integrity. Our results indicated a high correlation between NfL and YKL-40 in this sample and the effects of YKL-40 negated when tested in a model including NfL. Therefore, the next step would be to investigate to what extent these changes in axonal integrity and inflammation contribute to the vulnerability of the brain to neurodegeneration and how it reflects cognitive decline in normal and non-Alzheimer’s disease related aging of the brain. Future studies with longitudinal measurements of imaging and CSF could delineate the precise ordering of events and how the biological processes are intertwined. sTREM2, released by microglia, fluctuates over the course of Alzheimer’s disease, with an increase close to symptom onset.^28,29,58^ In the current study, the association between sTREM2 levels and grey matter networks disappeared in mutation carriers when analyses were corrected for age. We did not find compelling evidence that inflammatory processes due to microglial activity, as reflected by sTREM2 increases, may not be linearly related to grey matter morphological change.

A strength of the present study is that we evaluated the pathophysiology over the full course of Alzheimer’s disease. Investigating MCs from the DIAN study, along with NCs, is a powerful approach to investigate multiple disease processes that may contribute to grey matter network disruption. Due to the causative genes in dominantly inherited Alzheimer’s disease, the cross-sectional trajectory can inform longitudinal changes. Still, the reality is more complex,^59^ meaning further study in a longitudinal design is needed to understand the drivers and downstream effects in disease progression. A shortcoming of fitting Alzheimer’s disease biomarker trajectories over the estimated years to symptom onset is that results in part depend on sample sizes and model assumptions. All EYOs of divergence were similar to previous studies, except for Ng and YKL-40, which is an indication of the level of robustness across modelling methods.^27^ sTREM2 was estimated to change later compared to a recent large study but consistent with an older study with the same data.^28,30^ Of note regarding generalization of biomarker trajectories, estimated with cross-sectional data often diverse earlier compared to the estimates based on longitudinal data as there is a strong bias towards earlier years from cross-sectional data.^59^ For the interpretation and validity, this means that the EYO estimates have a value on a group level and inform on the sequence of changes, but do not often translate to the individual level. In addition, the exact meaning of biomarker levels is not fully understood, as we were unable to investigate brain tissue as part of this study. Another limitation is that the log transformation of the CSF markers improved the distributions, but not all markers (pTau181, tTau, Neurogranin and NfL) passed formal normality testing. This means that when we assessed linear relationships between CSF and grey matter network values, it may underestimate existing relationships. Still, most correlations look in the scatterplots linear, and we also conducted subgroup analyses to evaluate whether patterns depended on disease severity, which could give rise to non-linear patterns. Still, some of those disease stage groups were of small size, and larger samples are required to further investigate these relationships in detail. Lastly, in this study, we studied a primary summary measure of network organization in order to reduce the number of comparisons and increase the interpretability of the data. The findings warrant follow-up research to evaluate whether associations are specific for specific brain areas or network measures and their generalization to sporadic (late onset) Alzheimer’s disease.

To summarize, loss of synaptic integrity and, in particular, axonal integrity, as evidenced by increased NfL in CSF, appears to be related to disrupted grey matter network organization in ADAD. These findings suggest that normalization of neuronal injury or synaptic processes might lead to stabilization or improvement of grey matter network integrity.

## Supplementary Material

fcae357_Supplementary_Data

## Data Availability

The data from the DIAN study can be requested online at https://dian.wustl.edu/, accessed December 2023.
