# Supplementary material for: Axonal damage and inflammation response are biological correlates of decline in small-world values: a cohort study in autosomal dominant Alzheimer’s disease
Source: Brain Commun. 2024 Oct 9;6(5):fcae357. doi: 10.1093/braincomms/fcae357 (PMC11495221; doi:10.1093/braincomms/fcae357)
Supplement: fcae357_Supplementary_Data [file fcae357_supplementary_data.docx]

**Supplemental methods and materials**

**Supplementary Table 1. Available samples per group for associations with Grey matter network measures**

|  | Total N | NC | MC group 1 | MC group 2 | MC group 3 | MC group 4 |
| --- | --- | --- | --- | --- | --- | --- |
| Ab42, Ab40, pTau, tTau | 352 | 136 | 84 | 63 | 43 | 26 |
| SNAP | 330 | 126 | 77 | 60 | 41 | 26 |
| Ng | 331 | 126 | 77 | 61 | 41 | 26 |
| VILIP1 | 330 | 125 | 77 | 61 | 41 | 26 |
| NfL | 165 | 68 | 34 | 13 | 33 | 17 |
| YKL40 | 331 | 126 | 77 | 61 | 41 | 26 |
| sTREM2 | 164 | 68 | 34 | 13 | 33 | 17 |

Legend: NC = noncarrier, MC= mutation carrier. Aβ = Amyloid beta, pTau = phosphorylated Tau, tTau = total Tau, SNAP-25 = Synaptosomal-Associated Protein 25kDa, Ng = Neurogranin, NfL = Neurofilament Light, VILIP-1= Visinin-like protein 1, YKL40 = Chitinase 3-like 1, sTREM2 = soluble TREM2 relative to a reference sample.

**Supplementary Table 2. Association of CSF markers with the small world coefficient within mutation carriers (MCs) by severity groups**

|  | **Within MCs: interaction of group and CSF predictor** | | | | |
| --- | --- | --- | --- | --- | --- |
| **Predictors** | **Interaction**  **(F)** | **Ratio negative**  **(est slope)** | **Ratio positive & CDR 0**  **(est slope)** | **Ratio positive & CDR 0.5**  **(est slope)** | **Ratio positive & CDR 1-3**  **(est slope)** |
| **Aβ_42/40_ ratio** | 0.63; p=0.59 | 0.03 (-0.13,0.20); p=0.68 | 0.0 (-0.36,0.35); p>0.99 | 0.44 (-0.14,1.02); p=0.13 | 0.09 (-0.53,0.71); p=0.77 |
| **pTau** | 0.68; p=0.57 | -0.02 (-0.41,0.37); p=0.92 | -0.17 (-0.4,0.05); p=0.13 | ***-0.36 (-0.67,-0.06); p=0.02*** | -0.18 (-0.54, 0.19); p=0.34 |
| **tTau** | 0.34; p=0.8 | -0.04 (-0.36,0.27); p=0.78 | -0.13 (-0.35,0.1); p=0.27 | *-0.25 (-0.56,0.05); p=0.10* | -0.2 (-0.52,0.12); p=0.23 |
| **SNAP-25** | 0.36; p=0.78 | 0.06 (-0.19,0.31); p=0.64 | -0.03 (-0.24,0.17); p=0.75 | -0.14 (-0.43,0.16); p=0.36 | -0.07 (-0.34,0.2); p=0.61 |
| **Neurogranin** | 0.24; p=0.87 | 0.01 (-0.21,0.23); p=0.92 | -0.07 (-0.3,0.16); p=0.53 | -0.06 (-0.36,0.24); p=0.69 | -0.15 (-0.47, 0.17); p=0.35 |
| **NfL** | 0.16; p=0.92 | ***-0.77 (-1.36,-0.18); p=0.01*** | -0.51 (-1.17,0.14); p=0.12 | ***-0.67 (-1.04,-0.3); p<0.01*** | -0.50 (-1.26, 0.26); p=0.2 |
| **VILIP-1** | 0.30; p=0.83 | 0.09 (-0.15,0.33); p=0.46 | -0.06 (-0.26,0.15); p=0.58 | -0.02 (-0.3,0.26); p=0.89 | 0.02 (-0.25, 0.28); p=0.9 |
| **YKL-40** | 0.75; p=0.52 | *-0.22 (-0.45,0.02); p=0.07* | -0.11 (-0.35,0.13); p=0.37 | ***-0.46 (-0.84,-0.07); p=0.02*** | -0.18 (-0.66,0.3); p=0.46 |
| **sTREM2** | 0.82 ; p=0.48 | -0.13 (-0.57,0.32); p=0.58 | -0.07 (-0.5,0.36); p=0.74 | -0.07 (-0.52,0.38); p=0.76 | 0.36 (-0.15,0.86); p=0.17 |

Legend: CDR = clinical dementia rating scale, Aβ = Amyloid beta, pTau = phosphorylated Tau, tTau = total Tau, SNAP-25 = Synaptosomal-Associated Protein 25kDa, Ng = Neurogranin, NfL = Neurofilament Light, VILIP-1= Visinin-like protein 1, YKL40 = Chitinase 3-like 1, sTREM2 = soluble TREM2 relative to a reference sample. Linear model predicting the small world coefficient with the respective CSF biomarkers, adjusted for sex. We tested the interaction with disease severity. All CSF markers, except the ratio, are log-transformed.

**Supplementary Table 3. Estimated years to onset of divergence between mutation carriers and noncarriers**

|  | **EYO of divergence according 99% credible interval** | **EYO of divergence according 95% credible interval** | **EYO of divergence, according 99.5% credible interval** |
| --- | --- | --- | --- |
| **Grey matter network** |  |  |  |
| **Small world coefficient** | **-8** | -10.4 | -7.5 |
| **Traditional CSF markers** |  |  |  |
| **Aβ_42/40_ ratio** | **-17.8** | -18.4 | -17.6 |
| **Aβ_42_** | **-15.5** | -16.4 | -15.2 |
| **Aβ_40_** | **0.5** | -1 | 1.2 |
| **pTau** | **-17.7** | -19.1 | -17.2 |
| **tTau** | **-19.2** | -20.4 | -18.4 |
| **Emerging CSF markers** |  |  |  |
| **SNAP-25** | **-14.8** | -17.9 | -12.6 |
| **Ng** | **-19** | -20.1 | -18.2 |
| **NfL** | **-7** | -8.1 | -6.7 |
| **VILIP-1** | **-17.9** | -19.8 | -16.8 |
| **YKL-40** | **-7** | -10.1 | -6 |
| **sTREM2** | **-3.5** | -6.7 | -2.2 |

Legend: EYO = Estimated years to symptom onset, Aβ = Amyloid beta, pTau = phosphorylated Tau, tTau = total Tau, SNAP-25 = Synaptosomal-Associated Protein 25kDa, Ng = Neurogranin, NfL = Neurofilament Light, VILIP-1= Visinin-like protein 1, YKL40 = Chitinase 3-like 1, sTREM2 = soluble TREM2 relative to a reference sample. These analysis depend on sample sizes, which were for: small world N=439; Aβ42, Aβ40, pTau, & tTau N = 352; SNAP-25 & VILIP1 N=330, Ng & YKL-40 N=331, sTREM2 N=218; NfL N = 210. See also Supplementary Figure 12.

**Supplementary Table 4. Baseline values for biomarkers used for EYO in comparison to cross-modal data**

|  | **Noncarriers (NCs)** | **Noncarriers (NCs)** | **Mutation carriers (MCs)** | **Mutation carriers (MCs)** |
| --- | --- | --- | --- | --- |
| **Grey matter network** |  |  |  |  |
| **Small world coefficient** | 1.63 ± 0.05 | 1.62 ± 0.05 | 1.59 ± 0.09 | 1.59 ± 0.08 |
| **Traditional CSF markers** |  |  |  |  |
| **Aβ_42_ pg/ml** | 1,379 ± 464 | 1,407 ± 466 | 951 ± 635 | 974 ± 634 |
| **Aβ_40_ pg/ml** | 15,491 ± 4490 | 15,698 ± 4418 | 14,763 ± 4851 | 14,862 ± 4760 |
| **pTau pg/ml** | 14 ± 5 | 14 ± 5 | 32 ± 24 | 31 ± 23 |
| **tTau pg/ml** | 168 ± 56 | 169 ± 55 | 295 ± 169 | 290 ± 162 |
| **Ratio Aβ_42/40_** | 0.088 ± 0.010 | 0.089 ± 0.010 | 0.065 ± 0.034 | 0.066 ± 0.035 |
| **Emerging CSF markers** |  |  |  |  |
| **SNAP-25 pg/ml** | 3.6 ± 1.3 | 3.6 ± 1.3 | 4.6 ± 1.9 | 4.6 ± 1.9 |
| **Ng pg/ml** | 1,529 ± 736 | 1,563 ± 741 | 2303 ± 1186 | 2,297 ± 1,212 |
| **NfL pg/ml** | 820 ± 622 | 793 ± 544 | 1925 ± 1900 | 1,939 ± 1,762 |
| **VILIP-1 pg/ml** | 132 ± 52 | 133 ± 50 | 176 ± 78 | 174 ± 79 |
| **YKL-40 ng/ml** | 135 ± 66 | 133 ± 66 | 178 ± 92 | 173 ± 88 |
| **sTREM2, relative to reference sample** | 0.48 ± 0.22 | 0.47 ± 0.22 | 0.59 ± 0.29 | 0.58 ± 0.29 |

Legend: Aβ = Amyloid beta, pTau = phosphorylated Tau, tTau = total Tau, SNAP-25 = Synaptosomal-Associated Protein 25kDa, Ng = Neurogranin, NfL = Neurofilament Light, VILIP-1= Visinin-like protein 1, YKL40 = Chitinase 3-like 1, sTREM2 = soluble TREM2 relative to a reference sample. Light grey color are the values of table 1 for the main analysis of cross-modal comparison

**Supplementary**
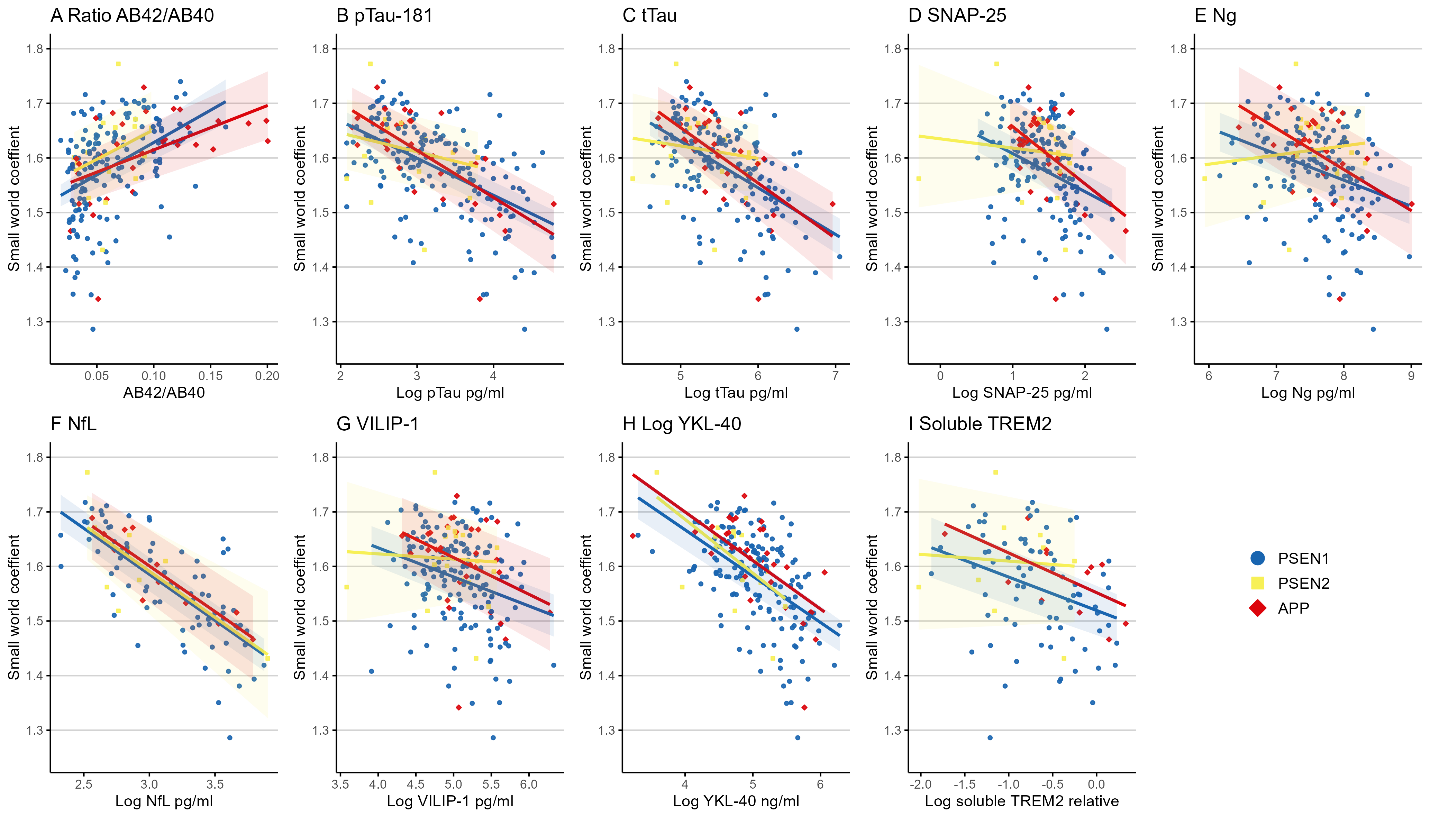
**Figure 1. Associations between CSF biomarkers and grey matter networks by mutation type**

Legend: Aβ = Amyloid beta, pTau = phosphorylated Tau, tTau = total Tau, SNAP-25 = Synaptosomal-Associated Protein 25kDa, Ng = Neurogranin, NfL = Neurofilament Light, VILIP-1= Visinin-like protein 1, YKL40 = Chitinase 3-like 1, sTREM2 = soluble TREM2 relative to a reference sample. Linear model predicting the small world coefficient with the respective CSF biomarkers, adjusted for sex. We tested the interaction with mutation type (linear model: lm(small world ~ biomarker level* mutation type + sex, data=dataset). None of the interactions with mutation type are significant (p<0.05). The graphs show the predicted values with 95% confidence intervals.

**Supplementary**
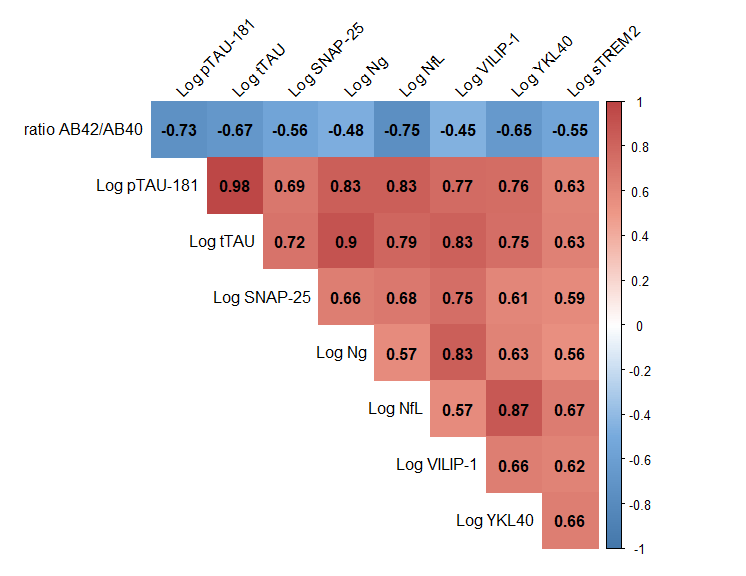
**Figure 2 Correlations between the CSF biomarkers**

Legend: Aβ = Amyloid beta, pTau = phosphorylated Tau, tTau = total Tau, SNAP-25 = Synaptosomal-Associated Protein 25kDa, Ng = Neurogranin, NfL = Neurofilament Light, VILIP-1= Visinin-like protein 1, YKL40 = Chitinase 3-like 1, sTREM2 = soluble TREM2 relative to a reference sample. The graphs shows the correlation coefficients for the CSF biomarkers in the dataset with all markers available (limited by NFL and sTREM samples size). All correlations are significant (p<0.05) and vary from R = -0.45 to 0.98.

**Supplementary Figure 3 Visualization of associations of NfL with other grey matter network metrics and traditional structural MRI measures**


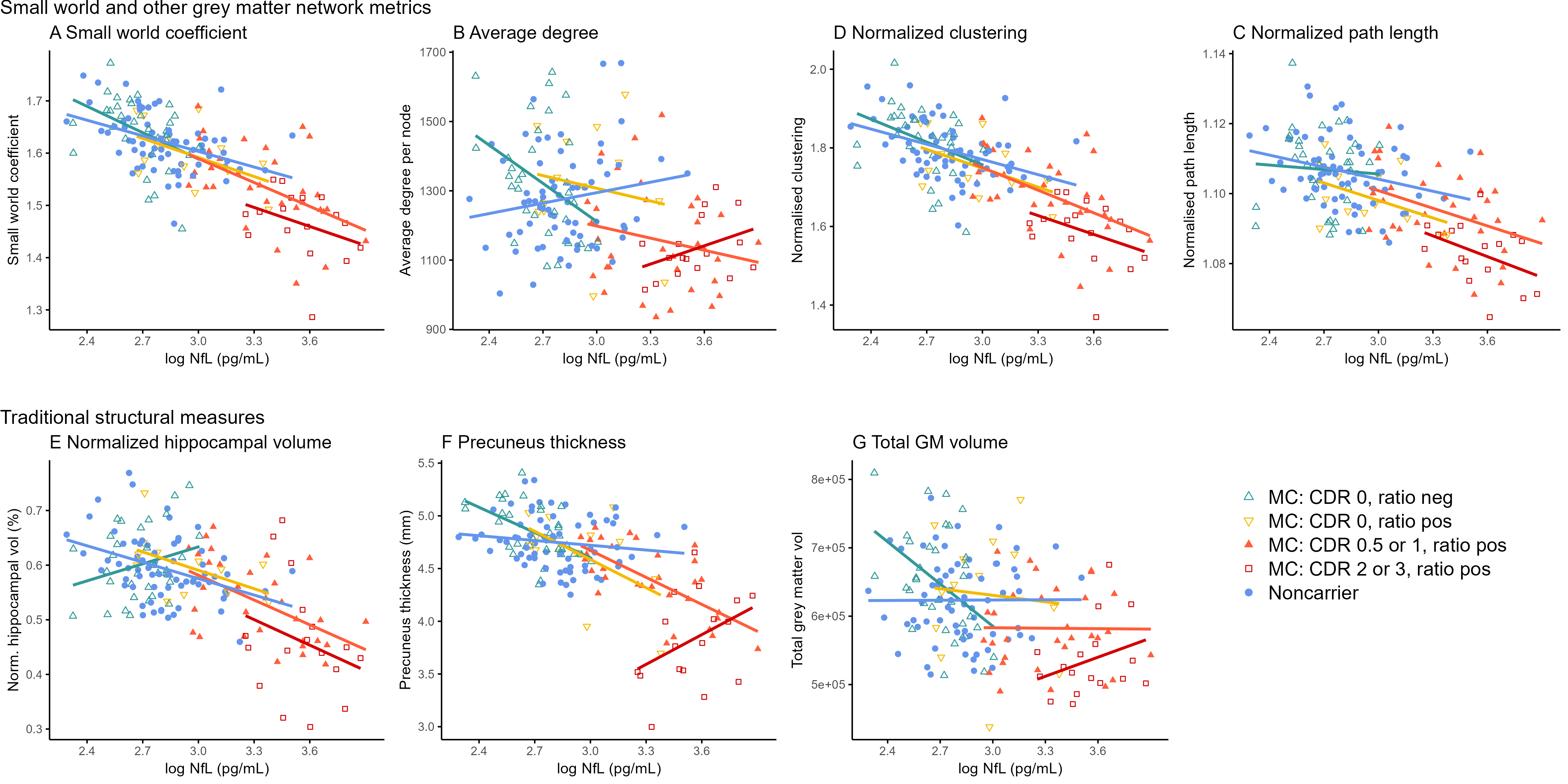


Legend: NfL = Neurofilament Light. MC= mutation carrier. CDR = clinical dementia rating. GM = grey matter. Line of linear models fitted for illustrative purpose. No statistical tests conducted.

**Supplementary Figure 4 Visualization of associations of YKL-40 with other grey matter network metrics and traditional structural MRI measures**


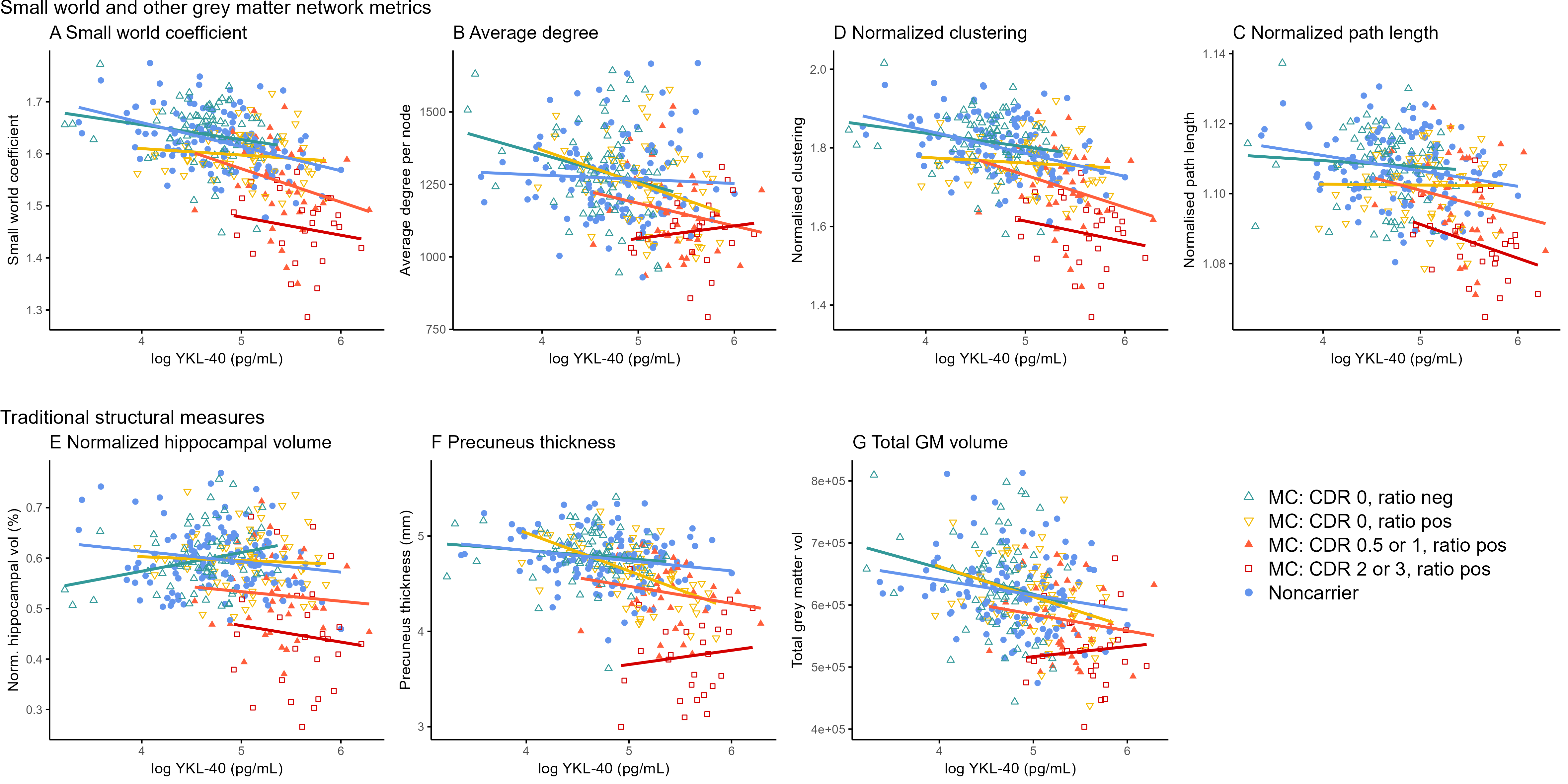


Legend: YKL40 = Chitinase 3-like 1. MC= mutation carrier. CDR = clinical dementia rating. GM = grey matter. Line of linear models fitted for illustrative purpose. No statistical tests conducted.

**Supplementary Figure 5 Visualization of associations of Abeta42/Abeta40 ratio with other grey matter network metrics and traditional structural MRI measures**


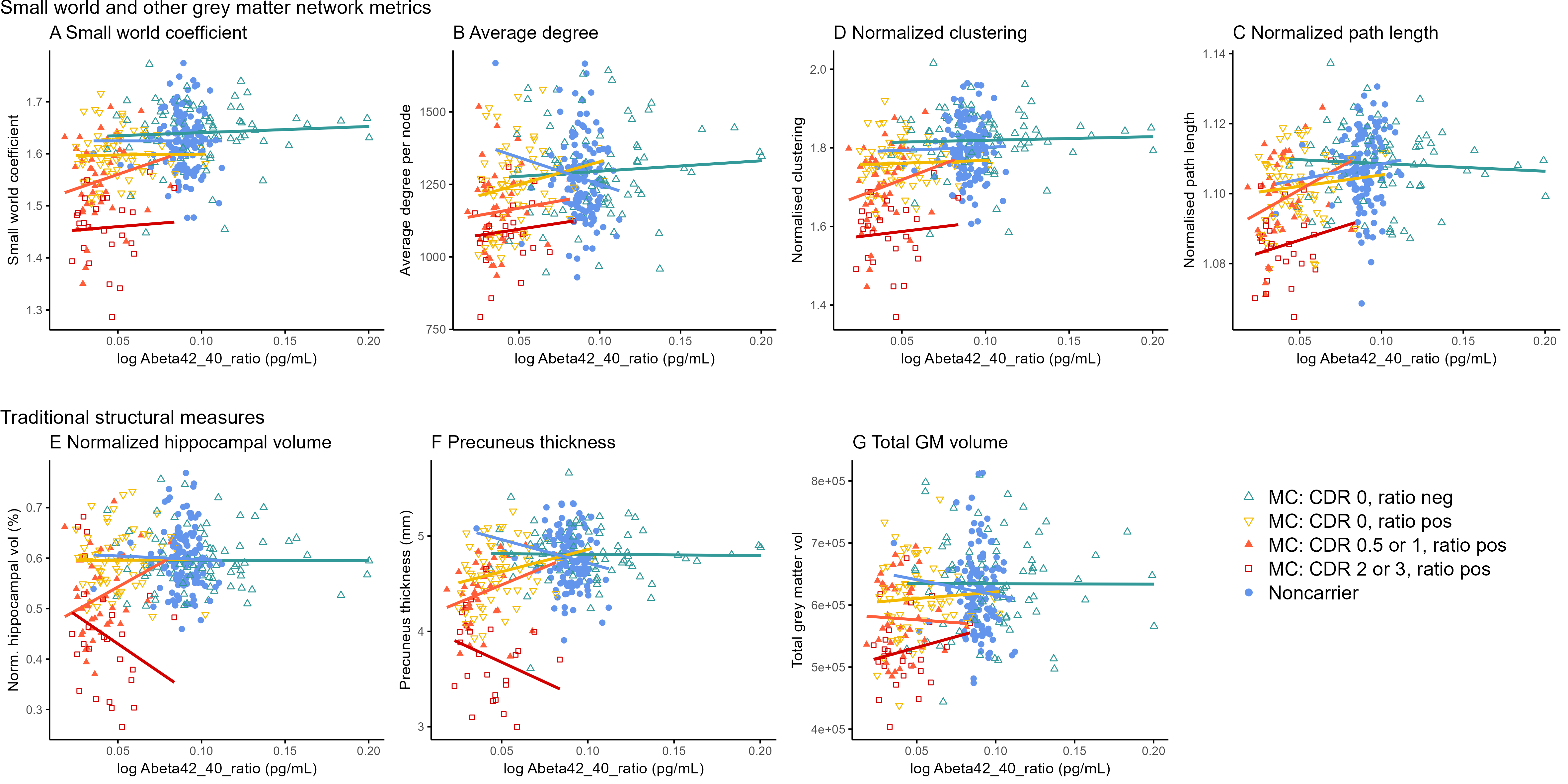


Legend: Aβ = Amyloid beta. MC= mutation carrier. CDR = clinical dementia rating. GM = grey matter. Line of linear models fitted for illustrative purpose. No statistical tests conducted.

**Supplementary Figure 6 Visualization of associations of pTau-181 with other grey matter network metrics and traditional structural MRI measures**


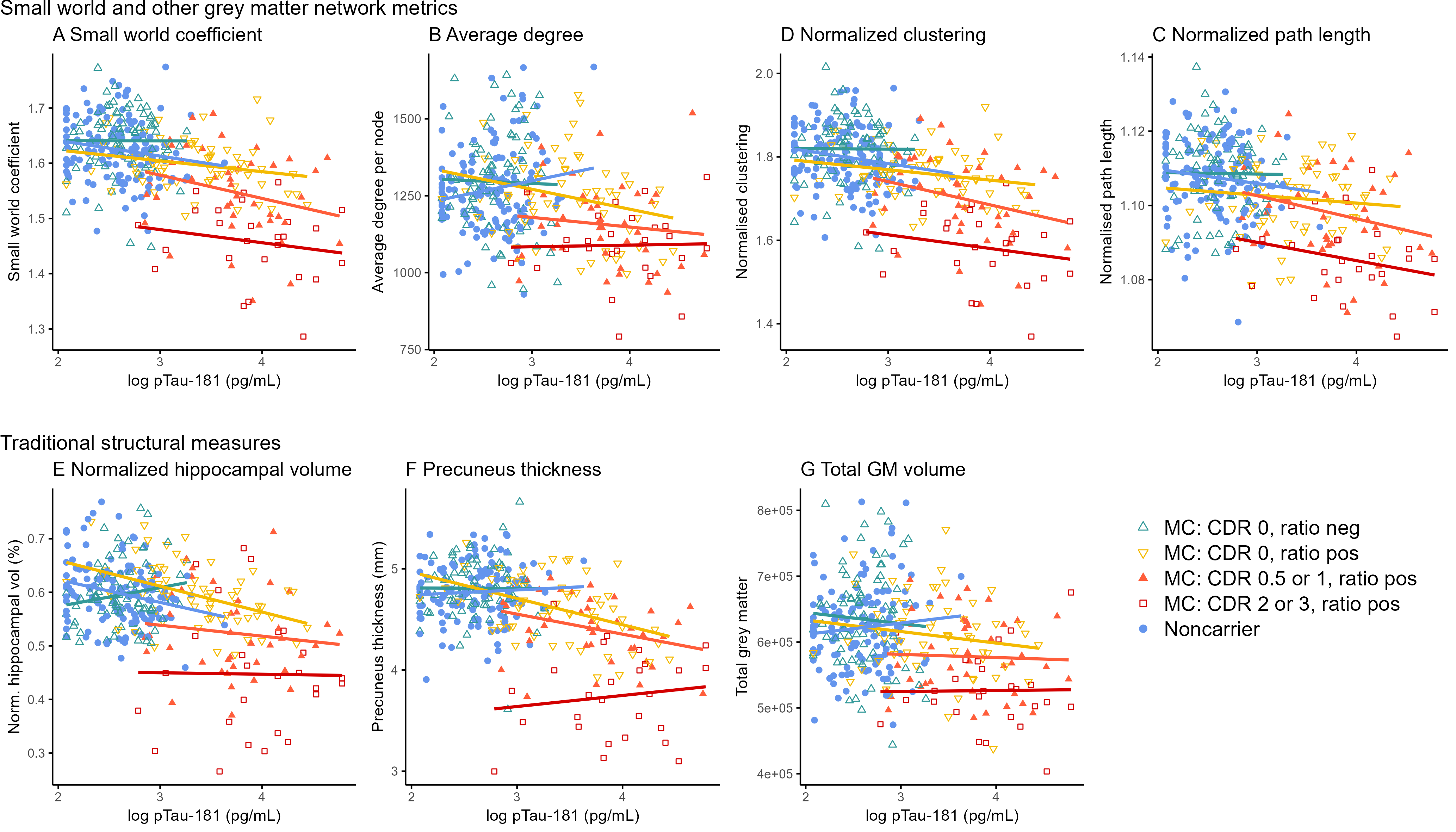


Legend: pTau = phosphorylated Tau. MC= mutation carrier. CDR = clinical dementia rating. GM = grey matter. Line of linear models fitted for illustrative purpose. No statistical tests conducted.

**Supplementary Figure 7 Visualization of associations of total Tau with other grey matter network metrics and traditional structural MRI measures**


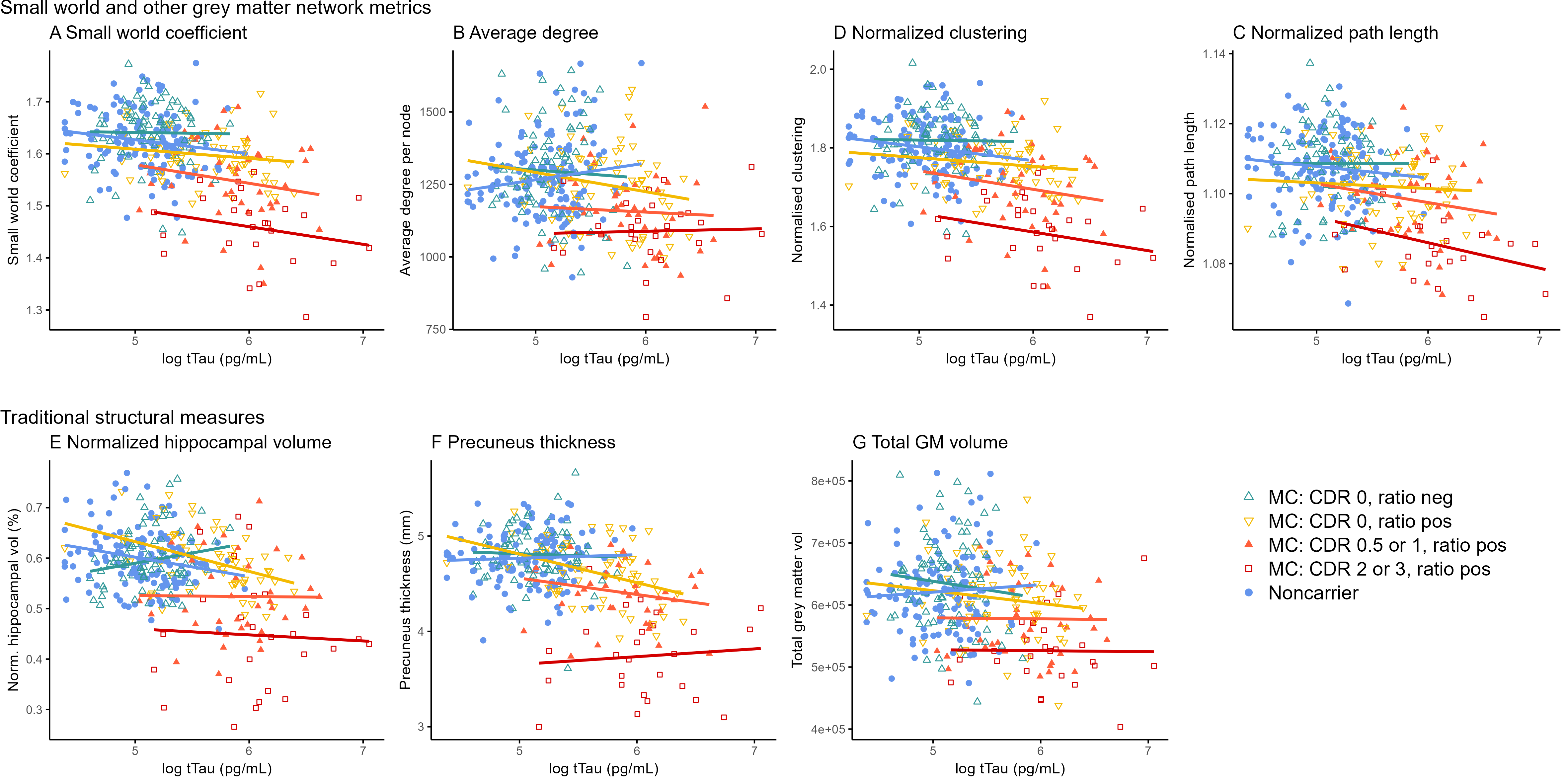


Legend: tTau = total Tau. MC= mutation carrier. CDR = clinical dementia rating. GM = grey matter. Line of linear models fitted for illustrative purpose. No statistical tests conducted.

**Supplementary Figure 8 Visualization of associations of SNAP-25 with other grey matter network metrics and traditional structural MRI measures**


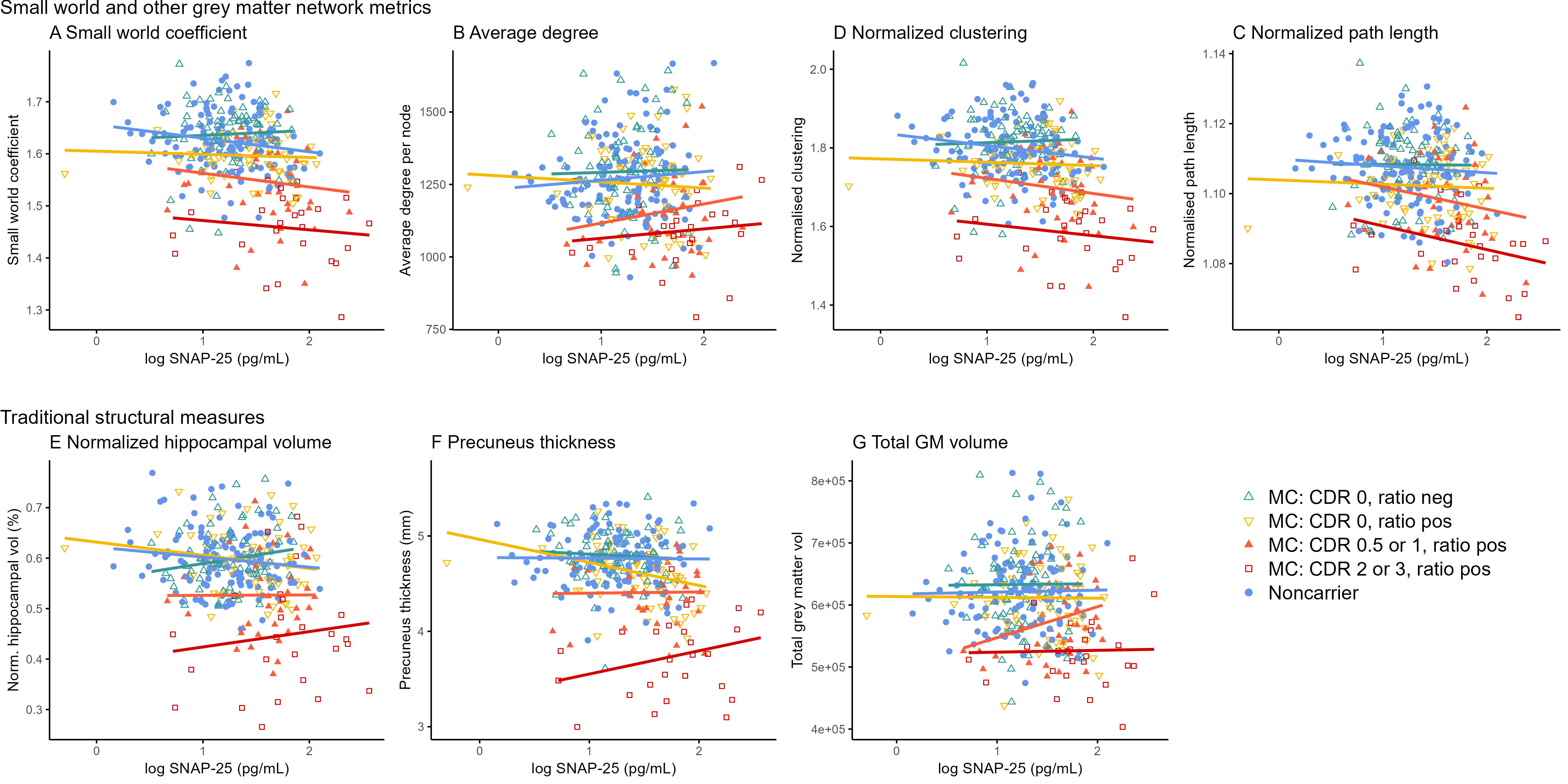


Legend: SNAP-25 = Synaptosomal-Associated Protein 25kDa. MC= mutation carrier. CDR = clinical dementia rating. GM = grey matter. Line of linear models fitted for illustrative purpose. No statistical tests conducted.

**Supplementary Figure 9 Visualization of associations of Neurogranin with other grey matter network metrics and traditional structural MRI measures**


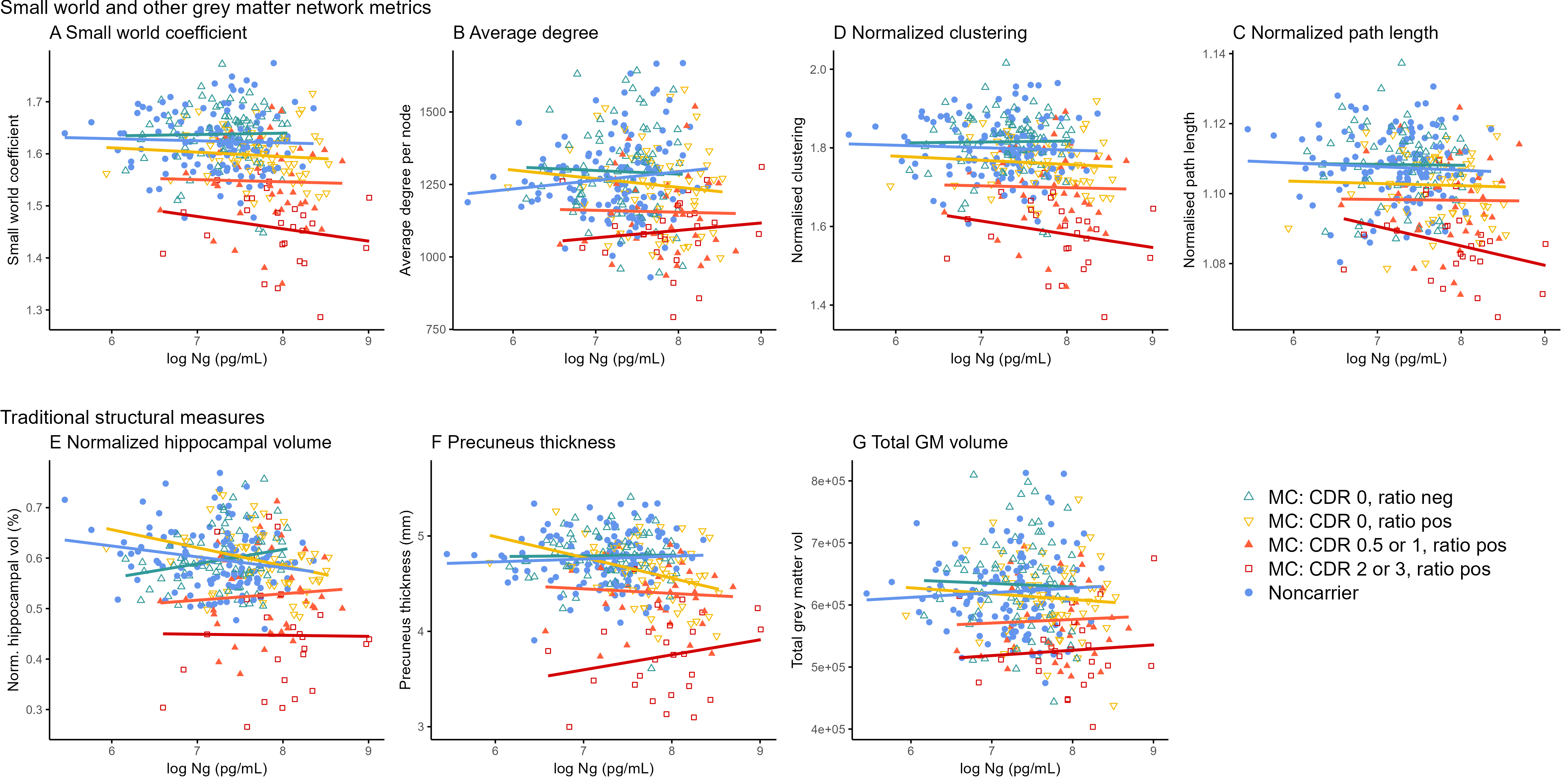


Legend: Ng = Neurogranin. MC= mutation carrier. CDR = clinical dementia rating. GM = grey matter. Line of linear models fitted for illustrative purpose. No statistical tests conducted.

**Supplementary Figure 10 Visualization of associations of VILIP-1 with other grey matter network metrics and traditional structural MRI measures**


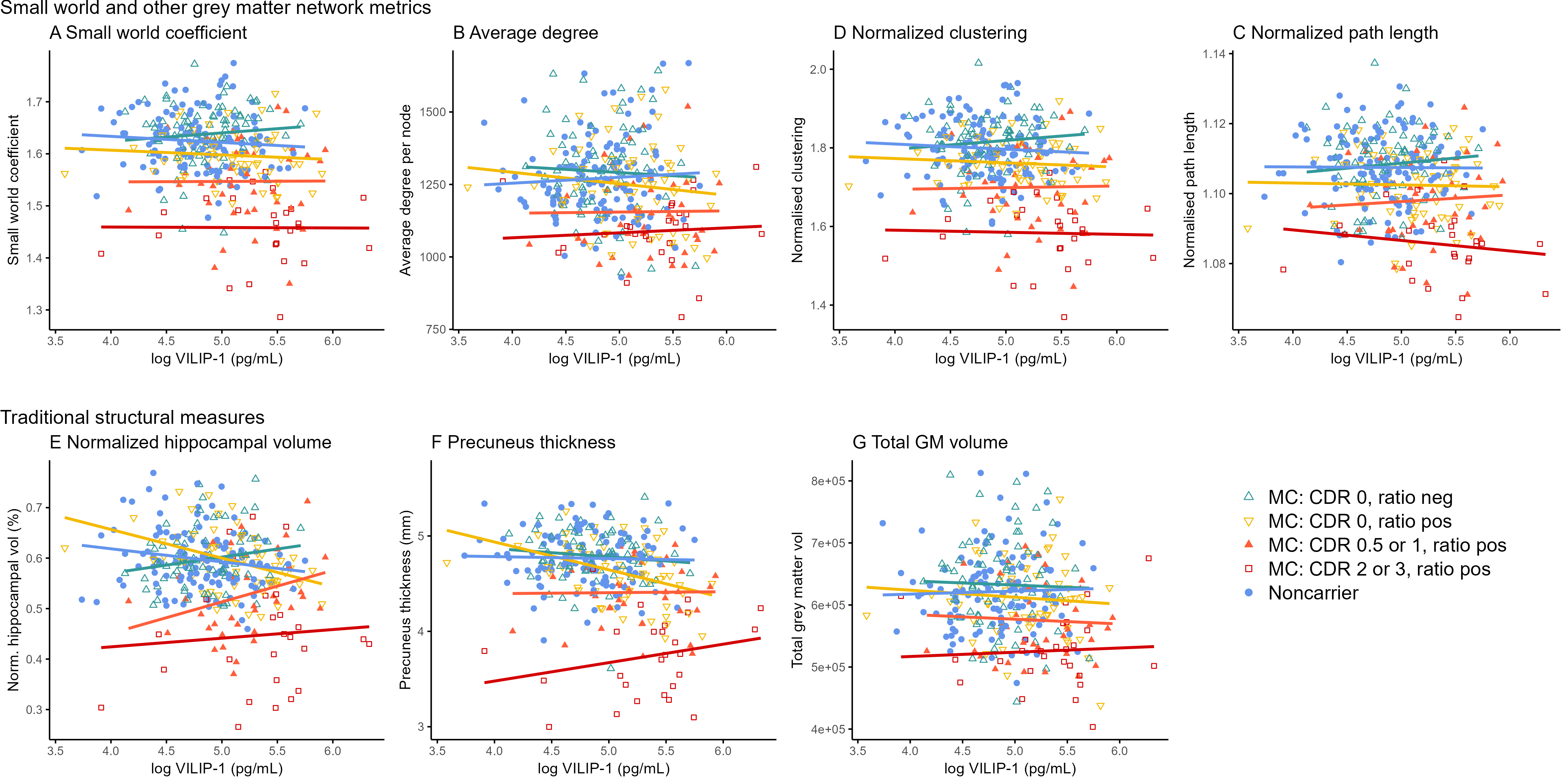


Legend: VILIP-1= Visinin-like protein 1. MC= mutation carrier. CDR = clinical dementia rating. GM = grey matter. Line of linear models fitted for illustrative purpose. No statistical tests conducted.

**Supplementary Figure 11 Visualization of associations of soluble TREM2 with other grey matter network metrics and traditional structural MRI measures**


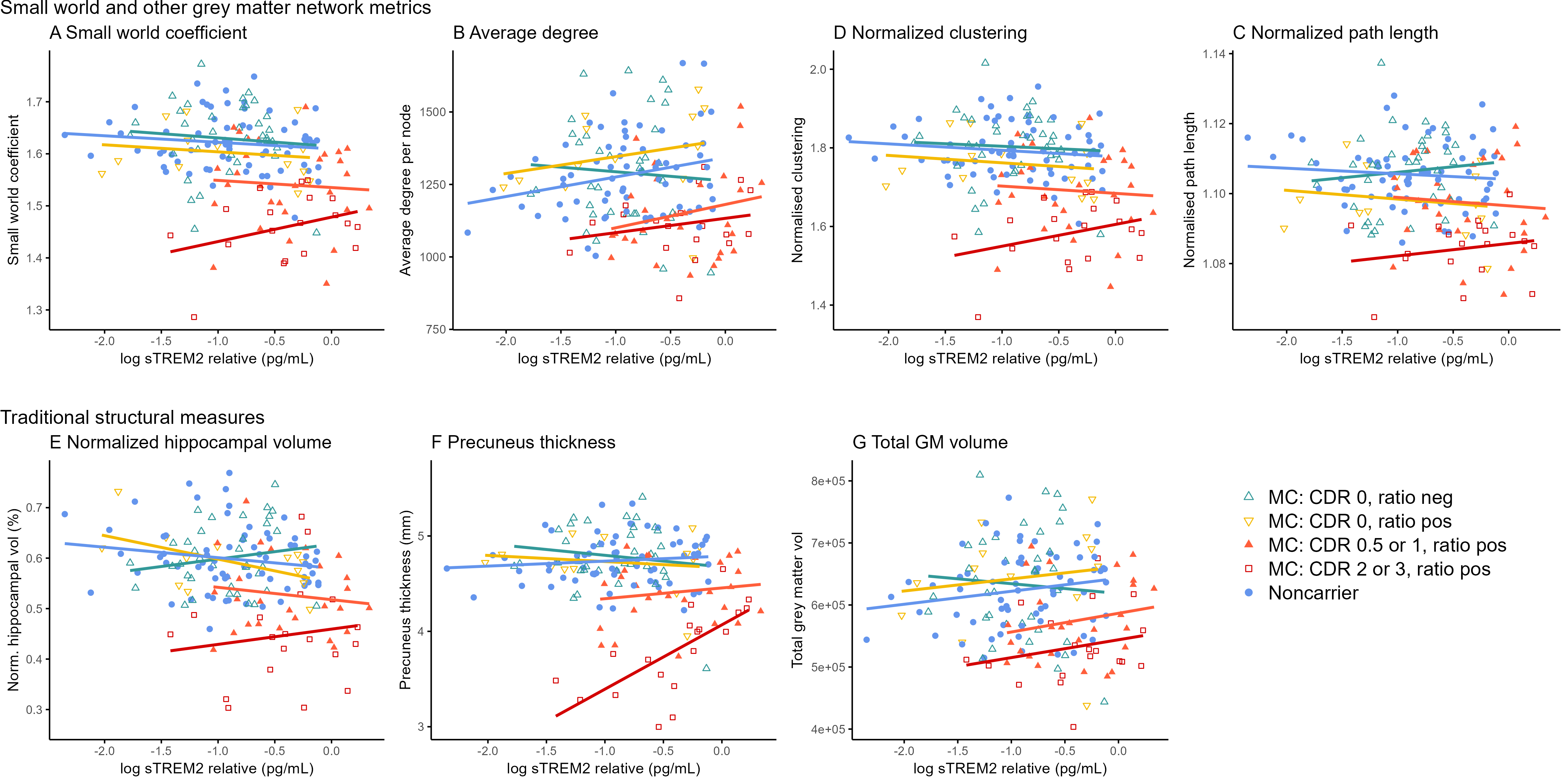


Legend: sTREM2 = soluble TREM2 relative to a reference sample. MC= mutation carrier. CDR = clinical dementia rating. GM = grey matter. Line of linear models fitted for illustrative purpose. No statistical tests conducted.

**Supplementary Figure 12. CSF and MRI biomarkers abnormality curves by EYO**


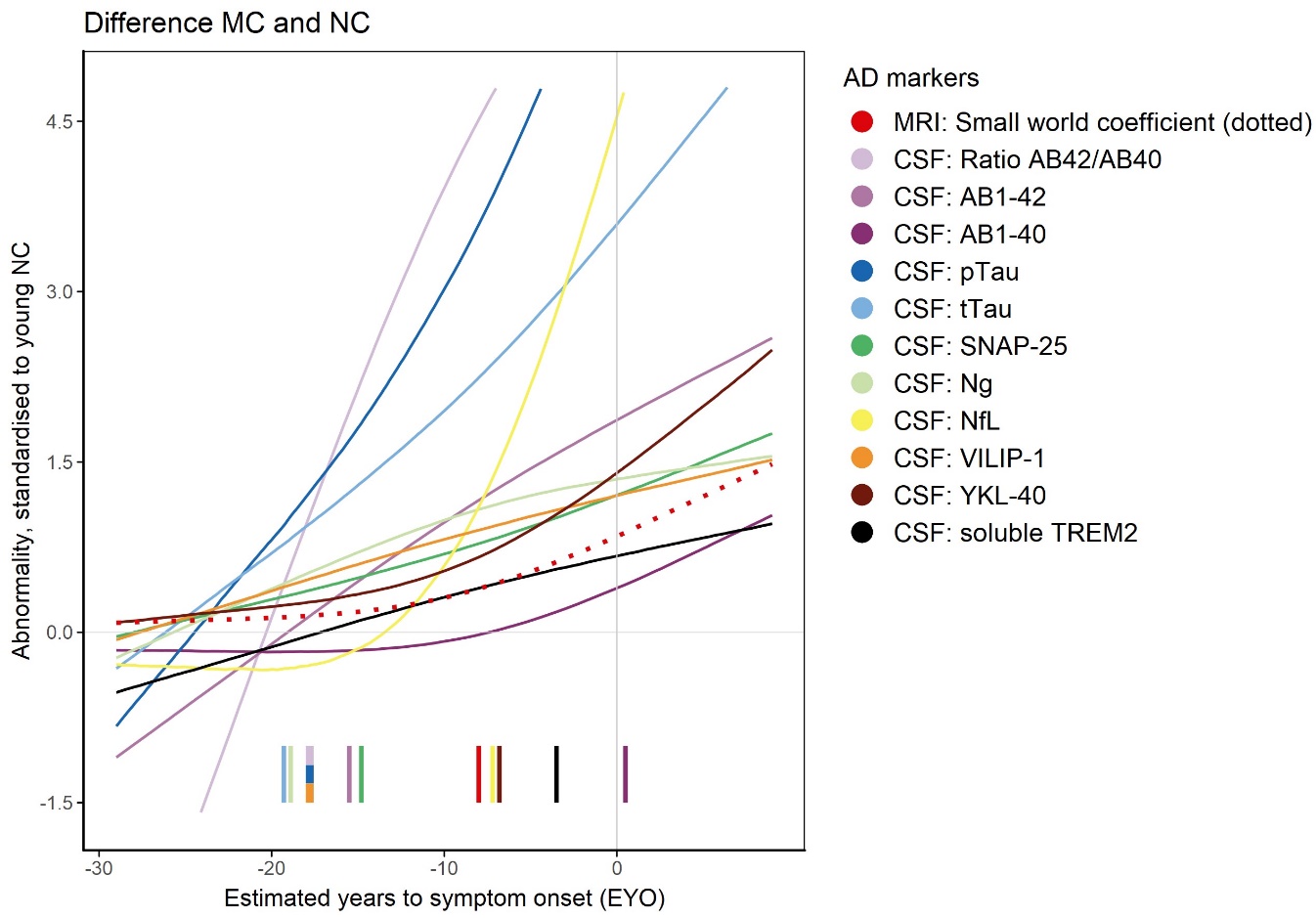


Legend: MC = mutation carrier, EYO = estimated years to symptom onset, CDR = clinical dementia rating scale. Aβ = Amyloid beta, pTau = phosphorylated Tau, tTau = total Tau, SNAP-25 = Synaptosomal-Associated Protein 25kDa, Ng = Neurogranin, NfL = Neurofilament Light, VILIP-1= Visinin-like protein 1, YKL40 = Chitinase 3-like 1, sTREM2 = soluble TREM2 relative to a reference sample. The graphs show the median estimated curves standardized to the noncarrier mean and standard deviation (Table 1). All fitted lines are the median of the mixed models with a cubic spline, family random intercept and sex as covariate, and for the small world coefficient also total grey matter volume. These analyses depend on sample sizes, which were for: small world N=439; Aβ_42_, Aβ_40_, pTau, tTau N = 352; SNAP-25, VILIP1 N=330, Ng and YKL-40 N=331; sTREM2 N=218; NfL N=210. The tickmarks are the point that the 99% credible intervals of the difference between mutation carriers and non-carriers is different than 0 (for EYO divergence points at different thresholds, see Supplementary Table 3).
